# Medicines in the Kitchen: Gender Roles Shape Ethnobotanical Knowledge in Marrakshi Households

**DOI:** 10.3390/foods10102332

**Published:** 2021-09-30

**Authors:** Irene Teixidor-Toneu, Sara Elgadi, Hamza Zine, Vincent Manzanilla, Ahmed Ouhammou, Ugo D’Ambrosio

**Affiliations:** 1 Natural History Museum, University of Oslo, 0562 Oslo, Norway; 2 Laboratory of Microbial Biotechnology, Agrosciences and Environment, Regional Herbarium ‘MARK’, Faculty of Sciences Semlalia, Cadi Ayyad University, Marrakech 40000, Morocco; sara.elgadi@ced.uca.ma (S.E.); hamza.zine@edu.uca.ma (H.Z.); ouhammou@gmail.com (A.O.); 3 Baseclear BV, 2333 Leiden, The Netherlands; Vincent.Manzanilla@baseclear.nl; 4 Global Diversity Foundation (GDF), 37 St. Margaret’s Street, Canterbury CT1 2TU, Kent, UK; ugo@global-diversity.org; 5 Etnobiofic Research Group, Institut Botànic de Barcelona (IBB-CSIC-ICUB), Universitat de Barcelona, 08038 Barcelona, Spain

**Keywords:** food–medicine continuum, functional foods, gastronomy, Marrakech, Morocco, pharmacopeia, spices, women’s knowledge

## Abstract

Differences in gendered knowledge about plants are contingent on specific cultural domains. Yet the boundaries between these domains, for example food and medicine, are sometimes blurred, and it is unclear if and how gender plays a role in creating a continuum between them. Here, we present an in-depth evaluation of the links between gender, medicinal plant knowledge, and culinary culture in Marrakech, Morocco. We interviewed 30 women and 27 men with different socio-demographic characteristics and evaluated how gender and cooking frequency shape their food and medicinal plant knowledge. We documented 171 ethno-taxa used in Marrakshi households as food, medicine, or both, corresponding to 148 botanical taxa and three mixtures. While no clear differences appear in food plant knowledge by gender, women have a three-fold greater knowledge of medicinal plants, as well as plants with both uses as food and medicine. Women’s medicinal and food plant knowledge increases with their reported frequency of cooking, whereas the opposite trend is observed among men. Men who cook more are often single, have university-level degrees, and may be isolated from the channels of knowledge transmission. This demonstrates that the profound relations between the culinary and health domains are mediated through gender.

## 1. Introduction

The boundary between foods and medicines is often more etic than emic, and it blurs when observing how plants are used in sociocultural contexts (e.g., [1,2]). Due to the plethora of antioxidants and phytonutrients present in the Plantae kingdom, phyto-nutraceuticals and folk functional foods are used not only as therapeutics but also as prophylactics, acting in preventing and inhibiting illness [3]. The prophylactic use of plants is documented in most traditional and local pharmacopoeias [4,5,6] as well as regional ones, as exemplified by the Chinese [7] and the European Pharmacopoeias [8,9]. In fact, many food supplements sold by pharmaceutical companies—natural or synthetic—derive their information from local knowledge and use of plants worldwide [3].

While food plants and medicinal plants have been often studied separately by ethnobotanists, the food–medicine continuum is gaining interest in recent years and has been the object of study in Africa [10], Europe [6,11,12], Asia [13,14], the Americas [5], and to a lesser extent Oceania. Studies range from ethnobotanical inventories and monographs to phytochemical analyses and regional reviews, all indicating the omnipresence of the food-medicine continuum across multiple floras, cultures, and contexts [15]. Most recently, a plethora of publications have appeared in relation to medicinal foods and COVID-19, where edible plants are used either as therapeutics or prophylactics [16].

In the Arabo-Muslim world, many medicinal plants used are common vegetables, fruits, or spices. In Mecca, Saudi Arabia, one third of the medicinal plants are common vegetable and fruit crops (e.g., onion, celery, cabbage, coriander, lemon, olive oil, and dates), and almost one-fifth are spices [17]. Spices such as fenugreek, cumin, aniseed, cinnamon, and ginger are also the most salient plants in Mecca [17]. Similarly, the medicinal plants that are most widely known and used among the Ksar Lakbir population (Rif Mountains, NW Morocco) are also used as spices and food supplements [18]. One-third of the plants used medicinally by the local population of the Tata province (S Morocco) are cultivated for food purposes, including dates, barley, coriander, parsley, and mint [19]. Fakchich and Elachouri [20] find that one-third of the medicinal plants used in the six Eastern Morocco provinces are added to food in at least one medicinal preparation. Beyond the double value of many plant products individually, the widespread use of ready-made mixtures and common plant combinations both in food and medicinal preparations may explain this overlap [18,21,22]. Mixtures can be functional foods and often have multiple ethnobotanical purposes [23]. Just like food condiments, teas may blur this distinction between edible and medicinal categories in Morocco, as in other parts of the globe [22,24]. Moreover, plants used medicinally to treat gastrointestinal problems are often also ingredients in traditional desserts [22], perhaps as a means to ease digestion after heavy meals, when these desserts are often served.

As is the case for many cultural domains, both healing and cooking are heavily gendered in Arabo-Muslim countries. A literature review conducted by Fakchich and Elachouri [20] indicates that women use medicinal plants more often than men in Morocco. Based on their own observations and further literature, the authors attribute this to women’s fondness for traditional practices and swifter transmission of knowledge about them, as well as to the prestige linked to herbal medicine practices in women’s social contexts [25]. Other studies show that women know more about medicinal plants in Muslim countries because they do most of the caring for family children and elders (e.g., [17,26]), which is in line with evidence from other parts of the globe (e.g., [27,28,29,30]). Women’s pivotal role as medicinal plant knowledge holders may stem from gendered labour tasks and spaces in many societies, which leads to differences in access to plant resources between men and women [29,30,31]. In Morocco, women manage most family food activities, which represent a major daily investment in time, energy, and know-how. In Moroccan culture, culinary knowledge is spread by word of mouth from mother to daughter, and men are advised to avoid the kitchen or face losing their virility in the eyes of males from the same household [32]. Thus, men and women’s knowledge differ in relation to the preparation of food. While the preparation and serving of food is mostly a women’s task, several dishes are only prepared by men (e.g., grilled meats or the typical Marrakshi slow-cooked meat dish of *tanjia*) [33].

Morocco is a diverse country at the geographical, ethnic, and social levels, with a culinary style that dates back hundreds of years. This plurality gives Moroccan gastronomy its aromatic flavours and clever and harmonious mixtures [32]. Morocco has been a transit zone on the spice route between the Spice Islands, the Far East, Central Africa, and Europe [34]. The use of spices is rooted in the rural traditions of Morocco’s indigenous Amazigh populations, as well as in the royal kitchens of the great Moroccan dynasties—the Almoravids, Almohads, Marinids, Saadians, and Alawites. Morocco was invaded by the Arabs in several phases from the 7th to the 14th century [35]. It inherited the high culinary culture of Baghdad during the time of the Abbasid caliphs, when this city was the capital of the Islamic Empire, and its luxury kitchen was influenced by Persian styles [34]. In 711, the Arabs invaded Spain, where they settled for nearly 800 years [35]. Until the expulsion of the Moors from Spain in 1492, there was a constant cultural exchange between Spain and Morocco [34]. A multicultural civilisation with people from various parts of the Muslim and Mediterranean world, including Jews and indigenous Christians, has remained since then. The Andalusians brought with them the friendly lifestyle that had developed in Spain and began a culinary renaissance. Sub-Saharan Africa also left its mark on Morocco, exchanging gold and other riches: the caravans that converged on the north included large numbers of women from Mali or Sudan who would often become cooks, as well as Ottoman influences through refugees from Algeria and Tunisia who emigrated while those countries were under Ottoman rule [32,34]. The central region of Morocco is the most culinarily diverse, particularly in the city of Marrakech. A refined and sophisticated bourgeois kitchen emerged in Marrakech. Marrakech’s Djemaa-el-Fna square is still today known as one of the most exciting places to eat in Morocco, and the city hosts many centuries-old spice markets.

Urbanisation is gradually reshaping Moroccan society, and cities are now emerging as an ideal venue for the development and propagation of new sociocultural models [36]. In cities, women, and especially younger women, have more opportunities to leave the household and assert their individuality through new economic and social activities. For example, in Jeddah (Saudi Arabia), single women knew significantly less medicinal plants than married women, but since medicinal plant knowledge also varied significantly with age, it is not possible to specify the driving factor of such difference [26]. Despite these changes, most women are still responsible for managing domestic work—having an outside job does not lighten their domestic workload, especially in the kitchen, where they continue to handle most of the work [36].

Urban kitchens and cooking are likely a key gendered space and task, leading to differences in medicinal plant knowledge between men and women in Arabo-Muslim countries. Given that many medicinal plants are also used in food preparation, gendered roles in cooking for the family might also contribute to their medicinal knowledge. Our goal is to evaluate how cooking influences the use and knowledge about medicinal plants among men and women in Marrakech. We hypothesise that women know more food and medicinal plants than men and that cooking more results in higher plant knowledge in both men and women. Our results will shed light on the role of gender in shaping the food–medicine continuum in an urban, Arabo-Muslim context.

## 2. Materials and Methods

To address our research question, ethnobotanical interviews with 30 women and 27 men of different marital status (married and not married) between 30 and 60 years old were carried out in Marrakech in September and October 2020. Given the gendered roles and social rules regarding interaction across gender in Muslim countries, men were interviewed by a male researcher (H.Z.) and women were interviewed by a female researcher (S.E.), facilitating participants to feel at ease and freely express themselves during interviews [37]. Each of these 57 participants were from a different household (i.e., we did not interview two people from the same household), and interviews were individual (Table S1). Informants were identified through snowball sampling, starting from our personal and professional networks [38]. A relationship of trust was established with informants through direct or indirect personal relations and continuous communication. During interviews, local COVID-19 regulations were followed: 1.5 m distance was always kept with informants, and masks were worn. Interviews with women took place mostly at their homes and with men in public spaces (e.g., cafes). Only one interviewee did not want to meet in person, and the interview took place by phone. The Code of Ethics of the International Society of Ethnobiology [39] was followed. Free, prior, informed consent (FPIC) was always sought prior to each interview: the project aims and methods were explained and a FPIC form prepared for participants to sign. Participants were anonymised by assigning them a code. Signed FPIC forms and participants’ personal data were kept under key and password at the Herbarium MARK (Cadi Ayyad University) until the article’s publication and deleted afterwards.

During interviews, we first collected data enabling us to describe the participant in terms of gender, age range, number of members in the household, profession, and neighbourhood. Secondly, we enquired about what plants the participant uses, noting down vernacular names and modes of use. After a first list was provided, we further enquired if other plants were used for specific uses, indicating possibilities such as tea, medicine, food, drinks, animal food or crafts, in order to get a list as comprehensive as possible. We further asked for detail regarding the medicinal use of food plants or gastronomic use of medicinal plants mentioned. Finally, we noted if the participant cooked and how often, for whom and what kinds of dishes he or she prepared (see the interview template in Table S2). Interviews were conducted in Moroccan Arabic, which was the preferred language of all participants. Interview answers were written down, transcribed, and translated to English, except for plant vernacular names, dish, and disease names, which were not translated. Transcription of Arabic words to Roman spelling follows the American Library Association and the Library of Congress Arabic Romanisation system [40].

We analysed our data at the level of item to preserve emic plant classifications. We define items by the different vernacular names given during interviews referring to single plant products, which may be sourced from the same botanical species or ethno-taxon. This captures the diverse food and medicinal uses of a single plant, distinguishing for example, the different uses of walnuts (dessert ingredient, called *grga’*) and walnut tree bark or wood (medicinal, called *swak*). Our interviewees sourced their plants and plant products from markets and shops, where adulteration is not uncommon and accurate botanical identification is challenging even when using DNA-based identification methods [41]. We identified putative botanical species based on ethnobotanical literature [21] (see Table S3). Latin plant names have been updated using The Plant List [42]. Ready-made plant mixtures used for cooking and healing are counted as one item, despite being mixtures of many different plants.

Per interview, we noted (1) the total number of plants, (2) the number of medicinal-only plants, (3) the number of food-only plants, and (4) the number of medicinal and food plants. Participants were asked how often they cooked and provided the number of times per day or per week when they prepare a meal (Table S1). According to these responses, frequency of cooking was defined as the number of times a participant cooked per week (varying from ‘not at all’ to ‘four times a day’, i.e., 28 times per week). 

We performed analysis of interviewee’s free-lists using cultural domain analysis. The free-list technique is used to elicit the elements of a cultural domain [43], in this case, medicinal and food plants. To do so, we used the Excel macro FLAME (Free-List Analysis under Microsoft Excel), which allows users to analyse data collected through free listing. Its creation was largely inspired from ANTHROPAC, which is a software developed by Borgatti [43]. After verifying and removing duplicate items from free lists, list analyses were performed including item’s salience (Smith’s index, which takes into account the frequency and position in which items are cited in free lists), frequency of citation (percentage of respondents that cite one item), and lists’ saturation (number of respondents necessary to list all cited items).

Descriptive statistics (mean, standard deviation) were used to summarise information about the diversity of responses that informants provided. We used Generalised Linear Models to explore if any of the socio-demographic variables or a combination of those explained differences observed in our results. Quasi-Poisson models were used given the overdispersion of the data [44]. Once the predictive value of all socio-demographic variables was analysed and the problem of multiple comparisons counteracted using a Bonferroni correction, we built a Generalised Linear Model taking into account interactions between predictive variables that had proved to significantly explain the number of items listed by participants. All analyses were implemented in R [45]. The R code and the data used for the analyses are available at https://github.com/vincentmanz/Morocco (accessed on 27 September 2021).

## 3. Results

Of the 30 women interviewed, 15 were married, 6 were single, 6 were divorced, and 3 were widowed. Of the 27 men interviewed, 12 were married and 15 were single. In total, our participants cited 171 vernacular names corresponding to approximately 148 different botanical vascular taxa and 160 items (including three ready-made mixtures), from 2516 citations in total. Our data saturation analysis shows that twenty respondents were enough to gather all the cited items with 37 respondents that did not add any further items (Table S4). Analysing women’s and men’s knowledge separately shows that saturation was reached with 16 and 15 respondents, respectively (Table S4). Thus, we are confident to have adequately captured the diversity of food and medicinal plants known and used in Marrakshi kitchens. Women cited more items than men in total as well as more medicinal plants specifically. Women listed 57 (±13) items on average of which 19% were only used medicinally, 38% were only used as food, and 43% had both food and medicinal uses. Men cited 31 (±9) items on average, of which 14% had only medicinal uses, 64% had only food uses, and 23% had both food and medicinal uses.

The number of items that women cite increases with frequency of cooking and decreases with level of studies (participants with university degrees cite significantly less plants than unschooled participants). Age or the number of co-habitants do not explain differences in the number of plants mentioned by participants. Older women cite more plants than middle-aged or young women (average number of total items cited respectively: 63, 54, and 49, but these differences are not statistically significant; Table 1). There seems to be no differences among men of different ages regarding the number of total items cited (average between 30 and 31 for all age groups). Women that are or have been married cite more plants than single women (59 in contrast with 48 on average, but these differences are not statistically significant; Table 1), and no differences are evident between single and married men.

**Figure 1:**
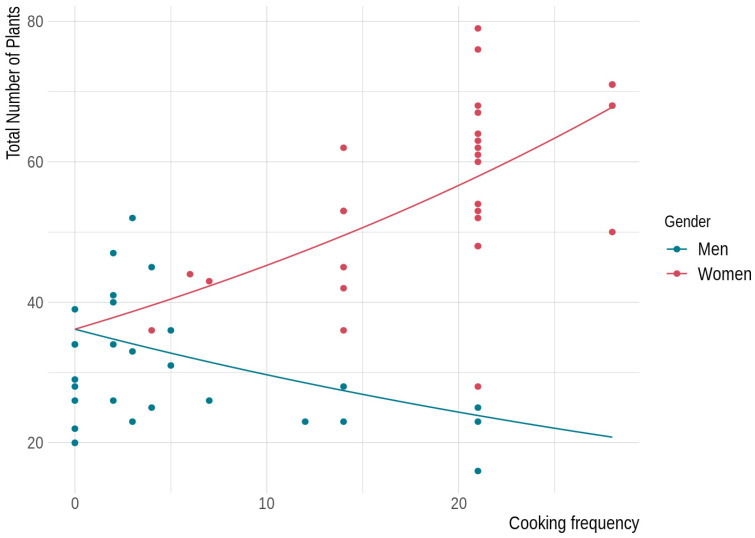
Number of elicited items according to frequency of cooking (number of times that the participant prepares a meal per week) per men and women. Trends are based on the GLM with interactions (see full model and results in Table S4).

The way in which frequency of cooking affects the number of items mentioned by participants is significantly contingent on gender (Table S5, Figure 1). Women usually cook for the whole family; they cook more when married, and cooking frequency increases with age. On the other hand, younger and unmarried men cook much more frequently than older and married men, but they cook mostly for themselves only. Single men cook daily, whereas married men cook twice a week, on average. All women interviewed cook at least four times per week, and no man cooks more than three times per day (Figure 1). Women cite more items than men, and the number of plants increases with the frequency of cooking, whereas the number of items cited by men decreases slightly when they cook more frequently (Figure 1). This is likely due to the fact that men that cook more are often single, live mostly on their own, have no children, and have university-level studies. The same trends are observed in regard to the number of medicinal plants and number of items used both as food and medicine but not regarding the number of items that are only used as food (Table 1 and Table S4). The number of items that are only food mentioned by participants does not change across any of the descriptive variables (Table 1).

One-hundred and two items were cited by more than five participants (Table 2). Men and women share knowledge of the most common cooking ingredients, but women often have unique medicinal knowledge about these same plants (Table 2). Some examples of items mentioned mostly as food by men and both as food and medicine by women include cinnamon (*qrfa*, *Cinnamomum verum* J. Presl), coriander (*qūzbr*, *Coriandrum sativum* L.), onion (*bṣla*, *Allium cepa* L.), and curcuma (*kharqūm*, *Curcuma longa* L.). Some medicinal plants are also exclusively cited by women, including *addad* (*Carlina gummifera* (L.) Less.), white horehound (*mrūta*, *Marrubium vulgare* L.), bitter cucumber (*ḥdja*, *Citrullus colocynthis* (L.) Schrader), and toothpick-weed (*bshnikha*, *Ammi visnaga* L.), among others. On the contrary, no medicinal plant is exclusively cited by men.

Some medicinal items only or mainly known by women are related to hair or skin treatments. For example, holm oak, rose, flax-leaved daphne, and henna. Other plants only cited by women are ingredients used in dessert recipes that only women prepare. These include peanut and sweet fennel, for example.

Whilst most plants are best known by women, some are almost exclusively cited by men. This is the case of okra, common bean, cauliflower, chickpeas, dates (*tmr*, fruit of *Phoenix dactylifera* L.), faba bean (*ful*, *Vicia faba* L.), and chili pepper (*hrūr*, *Capsicum annuum* L.) (Table 2 and Table S3).

The most salient items (Smith Index > 0.3) that contribute to the food–medicine continuum are spices: thyme, cumin seeds, ginger, garlic, saffron, clove, fennel seeds, cited as food and medicinal by both men and women; and cinnamon, oregano, and curcuma, cited mostly by women (Table 2 and Table S3). In comparison, fewer plants used in herbal teas are salient food–medicine plants (thyme, lemon verbena, rosemary, and pennyroyal). Three ready-made mixtures mentioned during interviews were *ras lhanūt*, *msakhn*, and *mrūziyah* (Table 2 and Table S3). *Ras lhanūt* was mentioned by men and women as used only as a flavouring for dishes such as *tajine*, which refers to the meat and vegetables cooked in an earthenware pot with the same name, or *rfissa* (Moroccan pasta called *trid* with chicken and lentils). *Msakhn* can also be used to flavour these dishes, but importantly, it is consumed medicinally by women during their menstruation or after childbirth and was only cited by women. *Mrūziyah* is a mixture used by men and women to flavour sweet meat and raisin dishes.

## 4. Discussion

Our study confirms women’s home caregiver role in Morocco. This role entails specific knowledge of plants and practices, including the provision of plant ingredients and the preparation of foods and medicines [28,29,30,31]. While our informants were not selected randomly and known and unknown biases may exist, our results confirm previous observations across Morocco. Women’s medicinal and food provisioning role and related knowledge is still widespread in the Arab world (e.g., [17,20,25,26]) as it was once important in the northern shore of the Mediterranean [46]. The observed similarities across gender in regard to food knowledge may also be due to men’s knowledge of the food items that they eat, which they do not necessarily prepare themselves. It is also important to note that women and men’s knowledge of food and medicinal plants is entangled through their distinct mobility and roles in public and private spaces. While women will prepare and serve foods and medicines, men are often responsible for the acquisition of local and exotic plant products in urban contexts. Thus, while men would know about plants used in the preparation of food through buying them and consuming them, women also have knowledge about the plants’ medicinal properties. Women’s more diverse medicinal knowledge may be primarily due to their role as family healthcare and food providers but also due to women-specific conditions and gender-specific culture. For example, women use medicinal foods to preserve health during pregnancy [10], and while the cultural domain of cosmetics in Arab countries is not exclusive to women, women do use more plants for a more diverse array of cosmetic practices, which is a trend that has been observed elsewhere [47]. In line with the socio-ecological theory of maximisation, already known plant resources would be used for a great diversity of purposes [48].

Gendered ethnobotanical knowledge has been also observed in the northern shore of the Mediterranean basin, which is a region that shares a common history, a relatively similar climate, and related floras and faunas with North Africa. Frazão-Moreira and Carvalho [49] observe similar gendered ethnobotanical patterns and conclude that gendered knowledge is highly related to the division of labour, tasks, and responsibilities of men and women. However, gender differences are not pervasive. In Southern Spain, wild plant collection is a gendered activity in some communities [50] but not others [51,52]. Gendered differences need to be understood in the larger socioecological context, including urbanisation characteristics, which are shown to drive these differences in Spain [52]. In Morocco, isolated hamlets in alpine areas maintain higher transmission of ethnobotanical knowledge across genders [53], diffusing the striking differences observed here in an urban context. Rapid urbanisation and the changing domestic roles that it entails, including readjustments in the *hadga* (the domestic attribute that refers to the skills and excellence in domestic chores supposed to characterise women) will likely impact ethnobotanical knowledge in the future. In Italy, changes in domestic roles with women’s integration into the paid workforce have resulted in women’s botanical knowledge loss [46].

The food–medicine continuum is expressed in Morocco through spices and recreational teas, as well as the perception that healthy foods contribute to the prevention of disease and promotion of health. Spices have been documented as flavouring ingredients and medicines since at least the Middle Ages [54]. Historical evidence shows that spices are added to food with the purpose of preserving health in the Islamic world [55], which is a feature that shows continuity with medicinal food in the Greco-Roman Mediterranean tradition [56]. Spices are often used in combination or as ready-made mixtures, which are more commonly used by women than men, as observed in Saudi Arabia [26]. On the contrary, wild leafy vegetables do not have a salient role as functional foods, which is typical of other cultures and contexts [57], perhaps because they are not as easily accessible in the city than in the countryside. However, wild leafy vegetables are not often used medicinally in at least one rural site close to Marrakech [58,59]. Recreational teas and other beverages seem to be less prominent than in European contexts [60,61], with spices taking the foreground. Islamic medicine promotes the preservation of health as more preferable than curing disease. Behaviours and preventive measures that contribute to maintaining health are recommended, among them, adequate food and drink [62]. Thus, the dual perception of foods as medicines and medicinal plants used in flavouring is embedded in the Islamic historical–religious context.

Leonti [63] argues that vegetables began to contribute to the human diet with the development of agriculture, which resulted in the distinction of gendered social roles that led to women taking responsibilities within the household [64,65]. Women might have been key to incorporating both food and medicinal plants to both the human diet and health praxis, thus developing the “food and medicinal continuum”. The strategic significance of wild food plants in ethnomedicine was overlooked by traditional ethnobotanical practice that did not take into account the gendered nature of knowledge and practice, because such medicinal foods are commonly harvested and consumed by women and children [66]. We echo long-standing claims that women’s inclusion in socio-ecological studies is key and can be achieved through the acknowledgement and valorisation of their knowledge, activities, and perspectives [30,37,67,68].

## 5. Conclusions

Our study corroborates that women know significantly more medicinal plants and plants with both medicinal and food uses than men in urban Arabo-Muslim contexts and shows that women’s ethnobotanical knowledge is concomitant to the cooking experience. No significant differences across genders were observed regarding the number of food plants known. This is likely due to the fact that the knowledge of food plants is not only acquired through the preparation of food but also through its acquisition and consumption. Men’s ethnobotanical knowledge does not increase with cooking frequency, which suggests a decoupling of the food and medicine knowledge systems among men. Age, number of co-habitants, and marital status had no effect on ethnobotanical knowledge, but participants with a university degree cited less plants than participants that had never attended school.

These results show the major contribution of women in linking knowledge between the food and medicine cultural domains. As we observed, women continue to play a key role in maintaining gastronomic and medicinal cultural heritage in present days. Under current global biocultural change, a better understanding of the links between food and medicine, men and women’s roles in cooking and healing, rural and urban connections and disconnections through plants, along with other past and present socioenvironmental dichotomies is crucial to refine and enhance the positive action in protecting cultures, local plant knowledge, and the environments they thrive in, depend on, and act upon.

## Figures and Tables

**Table 1:** *p*-values for the correlation between number of items listed per informant and socio-demographic descriptive variables (assessed through GLMs family Quasi-Poisson, Bonferroni corrected). Marital status *p*-values for divorced (D), married (M), and widowed (W) (single as model intercept), and level of studies’ *p*-values for primary (P), secondary (S), and university (U) (no schooling as model intercept). *p*-values < 0.05 are marked with an asterisk. “Med. & Food” items are those for which an informant mentioned both uses.

Number of Items	Gender	Age	Marital Status (D; M; W)	Level of Studies (P; S; U)	Number of Co-Habitants	Cooking Frequency *
Total	7.568 × 10^−11^ *	0.05324	0.34144; 0.08228; 0.33935	1; 1; 0.006193 *	1	3.806 × 10^−7^ *
Med. only	0.006193 *	0.1144	1; 1; 1	1; 1; 1	1	0.007755 *
Food only	1	1	1	1	1	1
Med. & Food	3.201 × 10^−11^ *	0.5093	0.5291; 0.1364; 0.3784	1; 1; 0.0010329 *	1	8.789 × 10^−7^ *

* Cooking frequency.

**Table 2:** Items cited by more than five participants, ordered according to the Smith’s saliency index. Number of citations indicates the number of participants that mentioned food (F), medicine (M), or food and medicine (FM) uses for each item, disaggregated by gender (w = women, m = men). Freq. indicates the frequency of citation in percentage.

Vernacular Name (English Name)	Scientific Name	Part Used	Number of Citations	Smith’s Index	Freq. (%)
Za’atar (thyme)	*Thymus vulgaris* L.	Leaves	w (28FM), m (19FM, 1F, 1M)	0.57	86
Kamūn (cumin)	*Cuminum cyminum* L.	Fruits	w (29FM), m (16FM, 7F)	0.56	89
Qūzbr (coriander)	*Coriandrum sativum* L.	Leaves	w (22FM, 3F), m (22F, 3FM)	0.54	88
Bṣla (onion)	*Allium cepa* L.	Bulbs	w (23FM, 8F), m (23F, 5FM)	0.53	96
Btata (potato)	*Solanum tuberosum* L.	Tubers	w (30F), m (26F)	0.53	98
Skinjbir (ginger)	*Zingiber officinale* Roscoe	Rhizomes	w (26FM), m (11FM, 8F)	0.49	79
Khizū (carrot)	*Daucus carota* L.	Roots	w (30F), m (26F)	0.48	98
Denjal (aubergine)	*Solanum melongena* L.	Fruits	w (30F), m (22F)	0.46	89
Tūma (garlic)	*Allium sativum* L.	Bulbs	w (23FM), m (14FM, 7F)	0.45	77
Na’ana’a, na’ana’a brūj (spearmint)	*Mentha spicata* L.	Leaves	w (18F, 10FM), m (15F, 3FM)	0.43	70
Z’fran (saffron)	*Crocus sativus* L.	Stamens	w (18FM, 3F), m (15FM, 4F)	0.42	68
Salmya (sage)	*Salvia officinalis* L.	Leaves	w (14M, 6F), m (12F, 3FM, 2M)	0.42	63
Ibzar (black pepper)	*Piper nigrum* L.	Seeds	w (20F), m (14F, 2FM)	0.41	63
Lwiza (lemon verbena)	*Aloysia citriodora* Palau	Leaves	w (23FM, 2F), m (13FM, 6F)	0.40	77
Ḥamḍ (lemon)	*Citrus limon* (L.) Osbeck	Fruits	w (23F, 9FM), m (15F, 6FM, 1M)	0.40	88
Z’itra (oregano)	*Origanum vulgare* L.	Leaves	w (16FM, 9M), m (6M, 5FM, 2F)	0.40	65
Khzāmá (lavender)	*Lavandula angustifolia* Mill.	Leaves	w (23M), m (16M)	0.38	67
Ma’dnūs (parsley)	*Petroselinum crispum* (Mill.) Fuss	Leaves	w (14F, 6FM), M (17F, 2 FM)	0.37	67
Azyr (rosemary)	*Rosmarinus officinalis* L.	Leaves	w (25FM), m (12FM, 2F)	0.37	68
Gar’a khadra, tikhrifint (pumpkin)	*Cucurbita pepo* L.	Fruits	w (30F), m (17F)	0.36	81
Felfla (bell pepper)	*Capsicum annuum* L.	Fruits	w (30F), m (8F)	0.35	67
Chiḥ (wormwood)	*Artemisia herba-alba* Asso	Leaves	w (19M), m (16M)	0.35	61
Gar’a hamra (winter squash)	*Cucurbita pepo* L.	Fruits	w (30F), m (17F)	0.35	82
Maticha (tomato)	*Solanum lycopersicum* L.	Fruits	w (30M), m (25F)	0.35	96
Qrfa (cinnamon)	*Cinnamomum verum* J.Presl	Bark	w (17FM, 1F), m (9F, 4FM)	0.35	72
Kharqūm (curcuma)	*Curcuma longa* L.	Rhizome	w (20FM, 2F), m (9F, 3FM)	0.35	60
Fliyū/Na’ana’a lfliwy (pennyroyal)	*Mentha pulegium* L.	Leaves	w (19FM, 11F), m (7F, 6FM)	0.33	61
Qrūnfl (clove)	*Syzygium aromaticum* (L.) Merr. & L.M.Perry	Flower buds	w (24FM, 4M), m (10FM, 1M, 1F)	0.33	65
Nafa’ (fennel seeds)	*Foeniculum vulgare* Mill.	Fruits	w (29FM), m (3F, 3FM, 1M)	0.32	60
Chiba (absinthe wormwood)	*Artemisia absinthium* L.	Leaves	w (22FM), m (9F, 2FM)	0.30	58
Zitūn (olive)	*Olea europaea* L. (fruit)	Fruits	w (18F, 5FM), m (15F, 2FM)	0.27	70
Ḥalba (fenugreek)	*Trigonella foenum-graecum* L.	Seeds	w (22FM), m (3FM, 2F)	0.26	47
Laft (wild radish)	*Raphanus raphanistrum* subsp. *sativus* (L.) Domin	Roots	w (28F, 2FM), m (5F, 1FM)	0.26	63
Ḥnna (henna)	*Lawsonia inermis* L.	Leaves	w (22M), m (3M)	0.25	44
Ḥab rshad (cress)	*Lepidium sativum* L	Seeds	w (17FM), m (4FM, 1F, 1M)	0.25	39
Khyar (cucumber)	*Cucumis sativus* L.	Fruits	w (30F), m (3F)	0.24	58
Mkhinza (Jesuit’s tea)	*Dysphania ambrosioides* (L.) Mosyakin & Clemants	Leaves	w (15M), m (11M)	0.23	46
Wrqt sydna mūsá (bay leaves)	*Laurus nobilis* L. (leaves)	Leaves	w (12FM, 3F, 1M), m (6F, 2FM)	0.23	42
Ward (rose)	*Rosa* sp.	Flowers	w (18M, 3FM), m (3M, 1FM)	0.22	40
Qa’qula, ḥbat hyl (cardamon)	*Elettaria cardamomum* (L.) Maton	Seeds	w (20F), m (2F, 2FM)	0.22	39
Khūs (lettuce)	*Lactuca sativa* L.	Leaves	w (30F), m (4F)	0.21	60
Timija (pineapple mint)	*Mentha suaveolens* subsp. *timija* (Coss. ex Briq.) Harley	Leaves	w (7FM, 5F), m (10F)	0.20	39
Ḥabat ḥlawa (anise)	*Pimpinella anisum* L.	Fruits	w (21FM), m (2FM, 1F)	0.20	39
Sanūj (nigella)	*Nigella sativa* L.	Seeds	w (20FM), m (4FM)	0.20	40
Bsibisa (mace)	*Myristica fragrans* Houtt.	Arillus	w (17F), m (3F)	0.19	35
A’tarcha (apple geranium)	*Pelargonium odoratissimum* (L.) L’Hér.	Leaves	w (12F), m (9F, 1FM)	0.18	33
Kaliptūs (eucalyptus)	*Eucalyptus* sp.	Leaves	w (16M), m (1M)	0.18	30
Krafs (celery)	*Apium graveolens* L.	Leaves	w (11F), m (11F)	0.18	39
Babūnj (chamomile)	*Chamaemelum nobile* (L.) All.	Flowers	w (17FM), m (1FM, 1M)	0.18	33
A’dss (lentils)	*Lens culinaris* Medik	Seeds	w (7FM, 2F), m (11F)	0.17	33
Tḥmira (chili pepper)	*Capsicum annuum* L. (powder)	Fruits	w (8F), m (9F)	0.17	28
Msakhn	Mixture 2	—	w (21FM)	0.16	37
Zari’t lkttan (flax)	*Linum usitatissimum* L.	Seeds	w (17FM), m (1FM, 1F)	0.16	33
Kamūn sūfi (woolly cumin)	*Ammodaucus leucotrichus* Coss.	Fruits	w (12M,1FM), m (2M)	0.15	25
Addad	*Carlina gummifera* (L.) Less.	Roots	w (13M)	0.15	23
Arq el-sūs (liquorice)	*Glycyrrhiza glabra* L.	Roots	w (9FM), m (2M, 2FM, 1F)	0.14	25
Ḥarmal (wild rue)	*Peganum harmala* L.	Seeds	w (13M), m (1M)	0.14	25
Badiana (Chinese star anise)	*Illicium verum* Hook.f.	Fruits	w (7M, 5F, 1FM), m (1M)	0.14	26
Mrdadūsh (marjoram)	*Origanum majorana* L.	Leaves	w (10F), m (3F, 1FM)	0.13	25
Na’ana’a al’bdy, a’abdya (peppermint)	*Mentha x piperita* L.	Leaves	w (11F), m (5F)	0.13	14
Limūn (orange)	*Citrus sinensis* (L.) Osbeck	Fruits	w (8F, 7FM), m (1F, 1FM)	0.13	28
Alzāz (flax-leaved daphne)	*Daphne gnidium* L.	Leaves	w (13M)	0.13	23
Gūza (nutmeg)	*Myristica fragrans* Houtt	Seeds	w (13FM), m (2F, 1FM)	0.12	28
Qūzbr yabs (coriander dried fruits)	*Coriandrum sativum* L.	Fruits	w (19FM)	0.12	32
Ḥbeq, lahbaq (basil)	*Ocimum basilicum* L.	Leaves	w (9F, 1FM), m (1M)	0.12	18
Kharūb (carob)	*Ceratonia siliqua* L.	Fruits	w (7FM, 5M), m (1M, 1F)	0.12	25
Zenjlan (sesame)	*Sesamum indicum* L.	Seeds	w (10F), m (7F)	0.11	30
Riḥan (common myrtle)	*Myrtus communis* L.	Leaves	w (9M), m (1M)	0.11	18
Zhar limūn (bitter orange flowers)	*Citrus × aurantium* L.	Flowers	w (9FM, 1M), m (1M)	0.10	19
Krwya (caraway)	*Carum carvi* L.	Fruits	w (9FM), m (3FM, 1M)	0.10	21
Illan (pearl millet)	*Pennisetum glaucum* (L.) R.Br.	Seeds	w (13FM), M (1F)	0.10	25
Lūz (almond)	*Prunus dulcis* (Mill.) D.A.Webb	Seeds	w (6FM, 3F), m (2F, 1FM)	0.09	21
Dbagh (holm oak)	*Quercus rotundifolia* Lam.	Roots	w (8M)	0.09	14
‘Ar‘ar (arar tree)	*Tetraclinis articulata* (Vahl) Mast.	Leaves	w (10M), m (2M)	0.09	21
Lūbya (common bean)	*Phaseolus vulgaris* L.	Seeds	w (2F), m (12F)	0.08	16
Mrūta, mariwa (white horehound)	*Marrubium vulgare* L.	Leaves	w (10M)	0.08	16
Sh’ir (barley)	*Hordeum vulgare* L.	Seeds	w (6F), m (2F)	0.08	14
Ḥdja (bitter cucumber)	*Citrullus colocynthis* (L.) Schrad.	Fruits	w (9M)	0.08	16
Ras lhanūt	Mixture 3	—	w (6F), m (3F)	0.08	12
Nwiwira (allspice)	*Pimenta dioica* (L.) Merr.	Seeds	w (8F)	0.08	14
Swak (walnut)	*Juglans regia* L.	Bark	w (11M), m (1M)	0.08	21
Slawi (calabash)	*Lagenaria siceraria* (Molina) Standl.	Fruits	w (3F, FM2), m (3F, 1M)	0.08	14
Kharwa’ (castor bean)	*Ricinus communis* L.	Seeds’ oil	w (7M)	0.07	12
Qchūr rman (pomegranate exocarp)	*Punica granatum* L. (Exocarp of the fruit)	Exocarp	w (9M), m (1M)	0.07	18
Trjla (common purslane)	*Portulaca oleracea* L.	Leaves	w (5FM, 3F), m (2F)	0.07	18
Jlbana (peas)	*Pisum sativum* L.	Seeds	w (2F), m (4F)	0.07	11
Bqūla, khābiza (cheeseweed)	*Malva parviflora* L.	Leaves	w (5FM, 1F), m (2F, 1FM)	0.07	16
Zit zitūn (olive oil)	*Olea europaea* L. (oil)	Fruits’ oil	w (2FM), m (4FM, 1F)	0.07	12
Sfarjl (quince)	*Cydonia oblonga* Mill.	Fruits	w (5F), m (4F)	0.06	16
Argan (argan)	*Argania spinosa* (L.) Skeels	Seeds’ oil	w (5FM), M (1F)	0.06	11
Nafa’ lbstany (sweet fennel)	*Foeniculum vulgare* Mill.	Fruits	w (8FM)	0.06	14
Kawkaw (peanut)	*Arachis hypogaea* L.	Seeds	w (7F)	0.06	12
Atay (green tea)	*Camellia sinensis* (L.) Kuntze	Leaves	w (5F), m (1F)	0.06	11
Fūwa (common madder)	*Rubia tinctorum* L.	Roots	w (5FM), M (2M)	0.05	12
Mkawar, kūrnb (wild cabbage)	*Brassica oleracea* L.	Leaves	w (2F, 1FM), m (6F)	0.05	18
Gamḥ (durum wheat)	*Triticum durum* Desf.	Seeds	w (5F, 1FM), m (1F)	0.05	12
Sūja (soybean)	*Glycine max* (L.) Merr.	Seeds	w (6FM)	0.05	11
Bshnikha (toothpick-weed)	*Ammi visnaga* (L.) Lam.	Inflorescences	w (7M)	0.04	12
Mlūkhya (okra)	*Abelmoschus esculentus* (L.) Moench	Fruits	w (1F), m (7F)	0.04	14
Khūdnjal (lesser galangal)	*Alpinia officinarum* Hance	Rhizomes	w (5F, 1FM), m (1F)	0.04	11
Nbag (jujube fruit)	*Ziziphus lotus* (L.) Lam.	Fruits	w (1F, 1FM), m (4M, 1F)	0.04	12
Shifrūl, chiflūr (cauliflower)	*Brassica cretica* Lam.	Flowers	m (6F)	0.02	11

## Data Availability

Data used for this study is available through this publication’s Supplementary Materials.

## Supplementary Materials

The following are available online at https://www.mdpi.com/article/10.3390/foods10102332/s1, Table S1: Socio-demographic and summarised interview responses per respondent, Table S2: Interview template in English and Arabic, Table S3: Item list including vernacular and Latin names, plant parts used, and detailed uses, Table S4: Saturation curves, Table S5: GLM code and results.
